# Single cell measurement of telomerase expression and splicing using microfluidic emulsion cultures

**DOI:** 10.1093/nar/gkv477

**Published:** 2015-07-21

**Authors:** Richard Novak, Kristina Hart, Richard A. Mathies

**Affiliations:** 1 UCSF/UC Berkeley Graduate Program in Bioengineering, University of California, Berkeley, CA 94720, USA; 2 Department of Chemistry, University of California, Berkeley, CA 94720, USA

## Abstract

Telomerase is a reverse transcriptase that maintains telomeres on the ends of chromosomes, allowing rapidly dividing cells to proliferate while avoiding senescence and apoptosis. Understanding telomerase gene expression and splicing at the single cell level could yield insights into the roles of telomerase during normal cell growth as well as cancer development. Here we use droplet-based single cell culture followed by single cell or colony transcript abundance analysis to investigate the relationship between cell growth and transcript abundance of the telomerase genes encoding the RNA component (hTR) and protein component (hTERT) as well as hTERT splicing. Jurkat and K562 cells were examined under normal cell culture conditions and during exposure to curcumin, a natural compound with anti-carcinogenic and telomerase activity-reducing properties. Individual cells predominantly express single hTERT splice variants, with the α+/β− variant exhibiting significant transcript abundance bimodality that is sustained through cell division. Sub-lethal curcumin exposure results in reduced bimodality of all hTERT splice variants and significant upregulation of alpha splicing, suggesting a possible role in cellular stress response. The single cell culture and transcript abundance analysis method presented here provides the tools necessary for multiparameter single cell analysis which will be critical for understanding phenotypes of heterogeneous cell populations, disease cell populations and their drug response.

## INTRODUCTION

Telomerase is a ribonucleoprotein complex that maintains telomeres at the ends of chromosomes through reverse transcription (1,2). Telomeres shorten with each cell division, and their maintenance is a key requirement for avoiding apoptosis. In humans, telomerase activity is present primarily during development and in stem cell and immune cell populations (2,3). Upregulation of telomerase is also observed in approximately 85–90% of all cancers, allowing cancer cells to avoid apoptosis despite uncontrolled cell division (4,5). The human enzyme consists of an RNA component, hTR, and a protein component, hTERT, in addition to other factors. hTR acts as a template for reverse transcription, and hTERT provides the catalytic activity as well as various binding sites for other proteins involved in telomere maintenance (2,6). In cancer cells, hTERT is the limiting factor, as hTERT is expressed from up to tens of mRNA copies per cell on average versus tens of thousands of hTR RNA molecules (7,8). In addition to regulating telomerase activity via hTERT levels, hTERT mRNA can be subject to alternative splicing that results in catalytically inactive protein (9). More than 20 alternative splice variants have been discovered, with only the full-length variant exhibiting telomerase activity (9,10). However, several studies have shown non-enzymatic roles for telomerase, including some of the alternative splice variants (9). Alpha and beta splice variants are the most frequently observed as well as the best studied, and both have been shown to inhibit telomerase activity (9,11,12). In the frequently used nomenclature, α+/β+ is the full-length hTERT mRNA, while splicing out of the alpha region (part of exon 6), beta region (exons 7 and 8), or both are referred to as α−/β+, α+/β− and α−/β−, respectively. This study examines only these most common four splice variants.

While telomerase and the roles of its splice variants have been extensively examined at the population or ensemble level using pools of presumably homogeneous cells, it is unknown how telomerase is expressed at the single cell level. This question is particularly important in the case of tumors, where accumulated mutations can result in highly heterogeneous cell populations (13–15). However, even healthy normal cells have been shown to exhibit high levels of heterogeneity and gene expression bimodality in response to stimuli (16). Understanding telomerase expression at the single cell level could clarify the role of alternative splice variants during cell division and colony formation. In particular, it is unclear from population-level studies, whether single cells produce only one or more splice variants, and what role if any alternative splicing plays in responding to environmental stimuli. These questions could be important in understanding cancer progression and in understanding the possible role of cell heterogeneity in therapeutic response.

Single cell culture with high throughput and normal cellular concentration is required to accurately determine meaningful cell division rates and to analyze telomerase in the daughter cells. Traditional single cell culture in titer plates is laborious and maintains cells at concentrations far below that of normal culture conditions, potentially introducing confounding effects due to altered environmental conditions. Alternatively microfluidic droplet generators have been used to rapidly produce picoliter to nanoliter droplets with high uniformity for digital polymerase chain reaction (PCR), single cell analysis and other applications requiring high throughput (17–21). Emulsions have also been used for encapsulating and culturing various cell types, though the systems have not demonstrated robust single mammalian cell culture due to the use of droplet volumes too small to accommodate significant cell growth (22,23). Development of a versatile single cell culture method that takes advantage of the parallelization and small volume scales of microfluidic emulsions is needed to provide a platform for studying multiple parameters of cell physiology and genetics.

**Figure 1.**
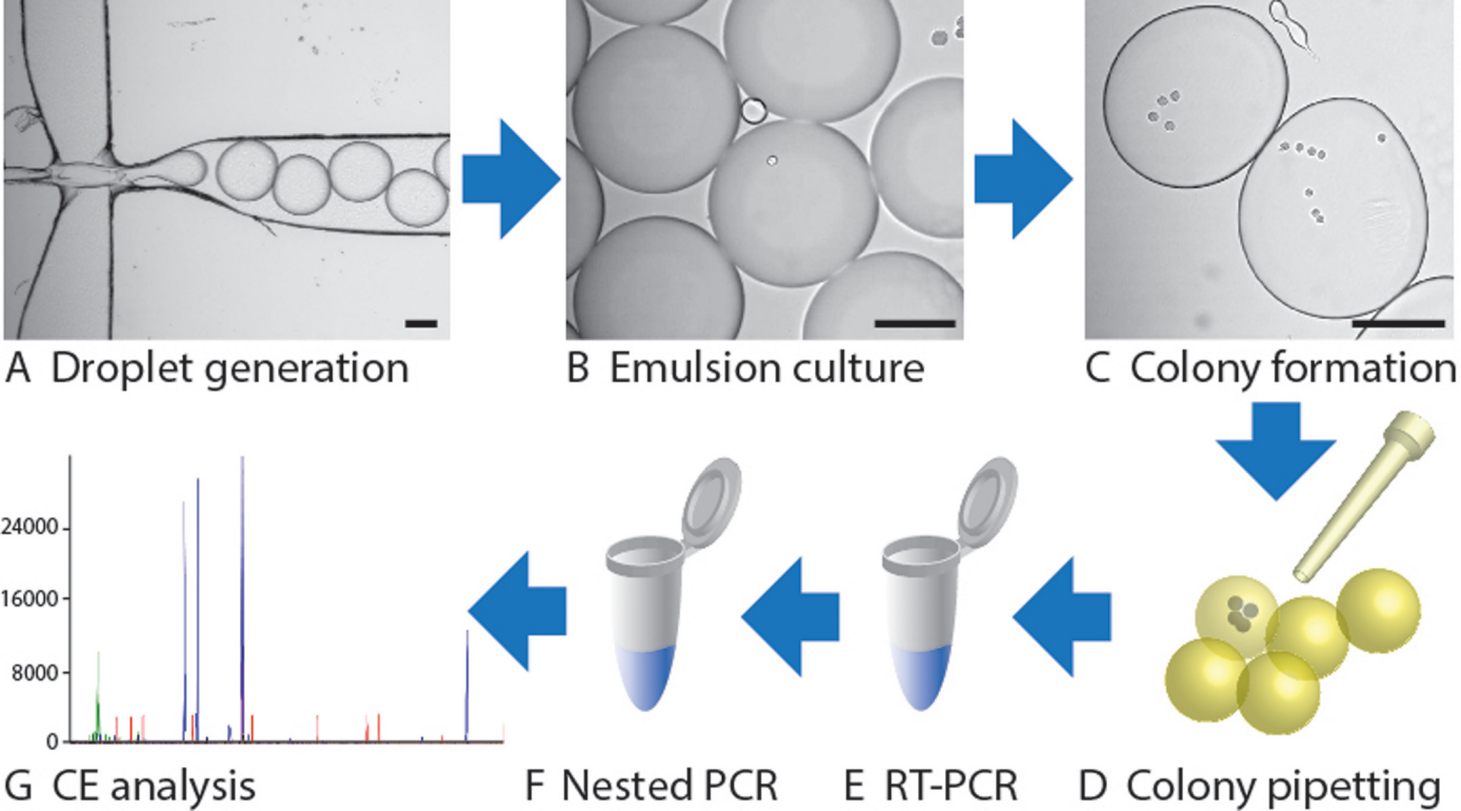
Workflow for single cell isolation, culture and analysis. Single cells are encapsulated in uniform 5.9 nl emulsions of culture medium using a microfluidic droplet generator (**A**) to form thousands of segregated culture “reactors” (**B**), where single cells can grow to form colonies (**C**). Individual encapsulated colonies formed from the founding cells are pipetted (**D**) into RT-PCR reactions (**E**) followed by nested PCR reamplification (**F**) for gene expression analysis of fluorescently labeled amplicons using capillary electrophoresis (CE) fragment analysis (**G**). Alternatively, colonies can be transferred to cell culture plates for subculture and further analysis. Scale bars are 100 μm.

Here we develop and characterize a microfluidic emulsion-based method for culturing thousands of single cells in parallel and apply it to understanding the relationship between telomerase transcript abundance and hTERT splicing and colony growth. Jurkat and K562 cells in culture medium are encapsulated using a custom microfluidic droplet generator, as shown in Figure 1, to form thousands of independent 5.9 nl compartments that are cultured in a standard incubator. The droplet cultures enable growth of cells at rates comparable to that of bulk cultures, while the compartmentalization and ease of manipulation permits the monitoring of colony size and subsequent correlation of cell growth rates with gene expression. We use an optimized reverse transcriptase-polymerase chain reaction (RT-PCR) assay with single molecule sensitivity followed by high-resolution fragment analysis of fluorescently labeled amplicons to probe telomerase transcript abundance and hTERT splicing patterns in native cultures. Finally, we investigate the impact of sub-lethal curcumin concentrations in cell culture on cell growth, transcript abundance and hTERT splicing as an example of how single cell expression analysis can be used to probe the mechanism of anticancer drugs.

## MATERIALS AND METHODS

### Microfluidic droplet generator fabrication

The droplet generator design (see Figure 1A) was created using AutoCAD (Autodesk), and a negative mold was cut into ScotchCal vinyl adhesive film (3M) using a Graphtec Craft Robo Pro CE5000-40 cutting plotter (Graphtec America) as described previously (24). The 125 μm high channels were transferred into a polystyrene Petri dish, and a polydimethylsiloxane (PDMS; 10:1 base:curing agent) device was cast from the sticker mold by pouring the PDMS mixture over the mold, degassing to remove trapped air bubbles and curing at 65°C overnight. Via holes were formed using a 0.75 mm biopsy punch (Ted Pella), and the approximately 5-mm thick device was bonded to a glass microscope slide following O_2_ plasma treatment and baked at 70°C for 5 min to facilitate adhesion. The channels were made hydrophobic by treating with 0.1% (heptadecafluoro-1,1,2,2-tetrahydrodecyl) dimethylchlorosilane (Gelest) in 100% ethanol for 10 min, followed by baking at 100°C overnight. Devices were reused by flushing with ethanol and distilled water.

### Cell encapsulation and culture

Droplets for cell encapsulation and culture were generated using an aqueous phase consisting of 0.5% Pluronic F68 (#P1300, Sigma-Aldrich), RPMI 1640 medium with L-glutamine (#11875-093, Gibco) supplemented with 20% fetal bovine serum (#16000-036, Gibco) and 1X PenStrep (#S108, Axenia Biologix). Cells were diluted to less than 0.1 cells per droplet in order to minimize the chance of co-encapsulation of multiple cells. Droplet Digital PCR fluorinated oil containing surfactant (Bio-Rad) was used as the continuous phase. Both liquid phases were actuated by a PHD 2000 infusion pump (Harvard Apparatus) with Hamilton syringes (gas tight #1001 for oil phase, gas tight #1750 for aqueous phase) at 20 and 30 μl/min flow rates, respectively, to produce 5.9 nl droplets. Syringes, tubing and chips were cleaned with ethanol and dried with N_2_ prior to each droplet generation session. Droplets were collected in 0.5 ml PCR tubes containing 250 μl oil. Encapsulated cells were incubated in a humidified atmosphere at 37°C and 5% CO_2_. Caps were punctured with a 1 mm biopsy punch to allow oxygen and CO_2_ flow to the cell colonies. Fluorinated oil allowed for diffusive passage of oxygen and CO_2_ in and out of droplets.

### Bulk cell culture

Jurkat and K562 cell lines were maintained at 37°C in a humidified 5% CO_2_ atmosphere in RPMI 1640 medium with L-glutamine (#11875-093, Gibco) supplemented with 10% fetal bovine serum (FBS, #16000-036, Gibco) and 1X PenStrep (#S108, Axenia Biologix). Cultures were maintained between 10^5^ and 10^6^ cells/ml. For bulk culture control experiments, FBS and cell concentrations were adjusted to those of droplet cultures. Cells in log growth phase were used for all experiments.

### Curcumin treatment

Curcumin powder (#C1386, Sigma-Aldrich) was dissolved to 10 mM in DMSO and stored at −20°C in single-use aliquots. Fresh working solutions were prepared by dilution in DMSO and added directly to cell cultures to a final concentration of 0.1% DMSO. Equal concentrations of pure DMSO were used for control cultures. Bulk cultures were maintained in 2 ml total volume in cell culture plates.

### Direct RT-PCR from colonies

Droplets were poured into a Petri dish containing a layer of emulsion oil, visualized at 10X magnification using a light microscope, and randomly sampled with a micropipette and MiniFlex gel loading tips (10 μl 0.17 mm inner diameter, #17360, Sorenson Bioscience). The colonies were deposited directly into 25 μl RT-PCR reactions containing 1X PCR Buffer (Invitrogen), 3 mM MgCl_2_, 0.2 mM dNTPs, 0.15 mg/ml heat-denatured bovine serum albumin (BSA; #A3803, Sigma-Aldrich), 5 mM DTT (Invitrogen), 1 unit/μl Superase In (Ambion), 10 units/μl SuperScript III (Invitrogen), 0.05 units/μl Platinum Taq (Invitrogen), 0.1% Triton X-100, 1 M betaine, 0.3 μM first round hTERT primers, 0.03 μM first round hTR primers and 0.03 μM first round GAPDH primers. To accommodate RNA targets with varying amounts of secondary structure, a reverse transcription step of 50°C for 15 min, 65°C for 1 min, cold block (−20°C) incubation for approximately 2 min, followed by additional reverse transcription at 25°C for 10 min and 50°C for 10 min was implemented. PCR amplification consisted of an initial heat activation step at 95°C for 2 min followed by 15 cycles of 95°C for 15 s, annealing at 62°C for 30 s, and extension at 72°C for 1 min, with a final 72°C extension step for 10 min. Primers were modified from the work of Yi *et al*. (7) and were purchased from Integrated DNA Technologies. See Supplementary Table S1 for sequence details. Primers for hTERT target only the region immediately surrounding the alpha and beta splice sites; therefore other less common splice variants outside of this region may be detected as full-length hTERT.

### Hemi-nested PCR

Reamplification reactions using hemi-nested primers contained 1X PCR buffer, 3 mM MgCl_2_, 0.2 mM dNTPs, 0.025 units/μl Platinum Taq (Invitrogen), 1 M betaine, 0.15 μM hemi-nested hTERT primers, 0.15 μM hemi-nested hTR primers, 0.075 μM hemi-nested GAPDH primers and 1 μl template from RT-PCR. Thermocycling consisted of an initial denaturation step at 95°C for 2 min followed by 35 cycles of denaturation at 95°C for 15 s, annealing at 62°C for 30 s, and extension at 72°C for 1 min, with a final extension step at 72°C for 10 min.

### Fragment sizing

To visualize and quantify fluorescently labeled amplicons, 0.5 ml of sample was added to 10 μl deionized formamide containing 2.5% GeneScan 500 ROX Sizing Standard (Applied Biosystems). Samples were analyzed on an ABI PRISM 3700 by the UC Berkeley DNA Sequencing Facility, and data were visualized and exported using Peak Scanner Software v. 1.0 (Applied Biosystems). Areas of target peaks were extracted using a custom MATLAB (The Mathworks) script and verified visually.

### Subculture of colonies

Single colonies at day 2 post-encapsulation were collected by pipetting and placed in round-bottom wells in 96-well plates containing 50 μl RPMI 1640 medium with L-glutamine (#11875-093, Gibco) supplemented with 20% fetal bovine serum (#16000-036, Gibco) and 1X PenStrep (#S108, Axenia Biologix) and maintained at 37°C in a humidified 5% CO_2_ atmosphere. Cultures were increased to 100 μl after 2 days and were supplemented with 50% fresh medium every 3–4 days.

### Data analysis

Peak areas for each target were normalized to cell number. Only reactions with GAPDH peaks were considered successful studies of live cells and were used in subsequent analyses. Plots depict means with standard deviation unless indicated otherwise. Hartigan's Dip Test was implemented in MATLAB (The Mathworks) and used to determine statistically significant deviation from unimodality (25,26). Of the available statistical tests and bimodality indices, this test has been found to most accurately determine multimodality (27). A two-tailed Student's *t*-test was used to assess growth rate differences between curcumin-treated and untreated cells. The Mann-Whitney U test (VassarStats) was applied to transcript abundance data for curcumin experiments, and threshold probability of *P* ≤ 0.01 and fold change >2 or <0.5 were used to determine statistical significance and biological significance, respectively.

## RESULTS

### Cell encapsulation and culture in microdroplets

To assess the growth rate of single cells at conditions comparable to standard cell culture, we utilized microfluidic droplet generators (μDG) to encapsulate single lymphocyte cells in segregated reactors. The cross-channel μDG nozzle design with continuous, syringe pump-driven flow of both oil and culture medium produced 3.4, 5.9 and 11.4 nl volume droplets at oil:medium flow rates of 30:20, 20:30 and 12.5:35 μl/min, respectively. Fluorocarbon oil with a biocompatible surfactant facilitated robust emulsions while being highly permeable to O_2_ and CO_2_. Cells were diluted to statistically dilute concentrations of less than 0.1 cells per droplet on average to minimize co-encapsulation of multiple cells in single droplets. The mean cell concentration was confirmed with microscopy.

**Figure 2.**
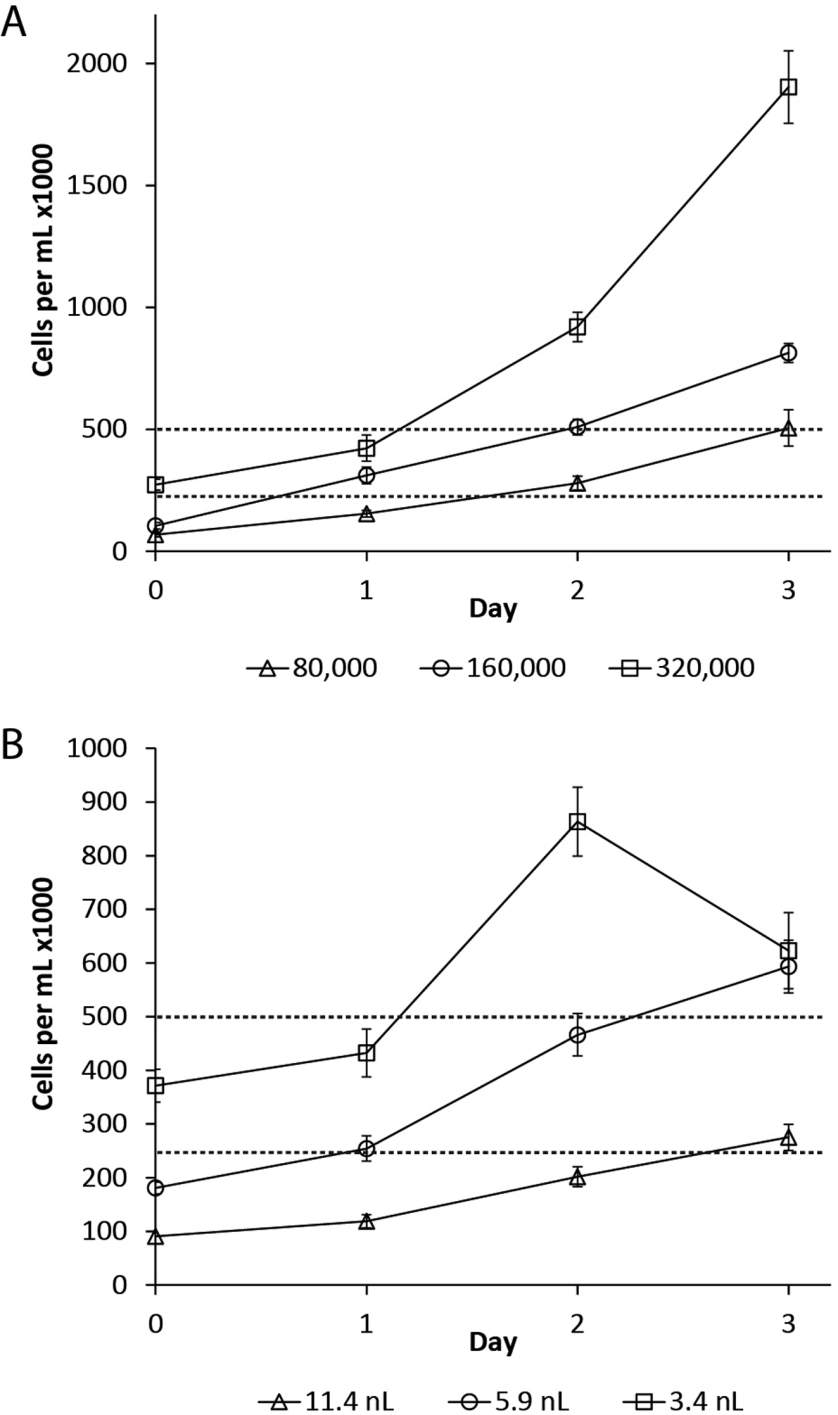
Comparison of the growth rate of Jurkat cells in bulk cultures (**A**) with encapsulated Jurkat cells (**B**) seeded at similar effective cell concentrations. Growth rates of Jurkat T cells in 3.4 and 5.9 nl droplets compare favorably with bulk cell cultures at equivalent starting cell concentrations of 80 000 to 320 000 cells per ml. However, the smallest droplets (squares) do not have adequate nutrients to maintain larger colonies after two days. The large 11.4 nl droplets (triangles) result in extremely slow growth rates. As a result, 5.9 nl droplets (circles) were selected for subsequent experiments. Mean ± standard error shown. Horizontal dashed lines at 250 000 and 500 000 cells/ml provide a visual guide for comparing the two plots.

The droplet volumes were selected in order to achieve single cell growth rates comparable to that of bulk cultures. Specifically, single cells in 3.4, 5.9 and 11.4 nl droplets correspond to approximately 87 000, 170 000 and 300 000 cells/ml, which spans the lower concentration range when passaging lymphoblasts. Due to the digital nature of encapsulation, the effective cell concentration in droplets with single cells relies solely on droplet volume and further dilution of cells only increases the number of empty droplets. Figure 2 compares the growth rate of Jurkat cells in bulk cultures (Figure 2A) with encapsulated Jurkat cells (Figure 2B) seeded at similar effective cell concentrations. Encapsulated Jurkats paralleled the growth trajectory of bulk cell populations at least until day 2 when nutrients are used up in the 3.4 nl droplets, as evidenced by the sharp decline in cell concentration on day 3 for the smallest droplet size. The starting cell concentration has a significant effect on the average growth rate for several days, with higher starting cell concentrations in both bulk and droplet cultures showing shorter doubling times than low starting concentrations. Unlike the encapsulated cells grown in 5.9 nl droplets, cells in 11 nl droplets failed to grow at rates comparable to those of bulk cultures; the largest droplet size was therefore not selected. In addition, the distribution of colony size versus day of culture shown in Supplementary Figure S1 further supported the finding that small droplets did not provide sufficient nutrients for the duration of the experiments. We therefore selected 5.9 nl droplet volumes for subsequent experiments.

### Multiplex RT-PCR assay for hTR, hTERT and GAPDH relative quantitation

**Figure 3.**
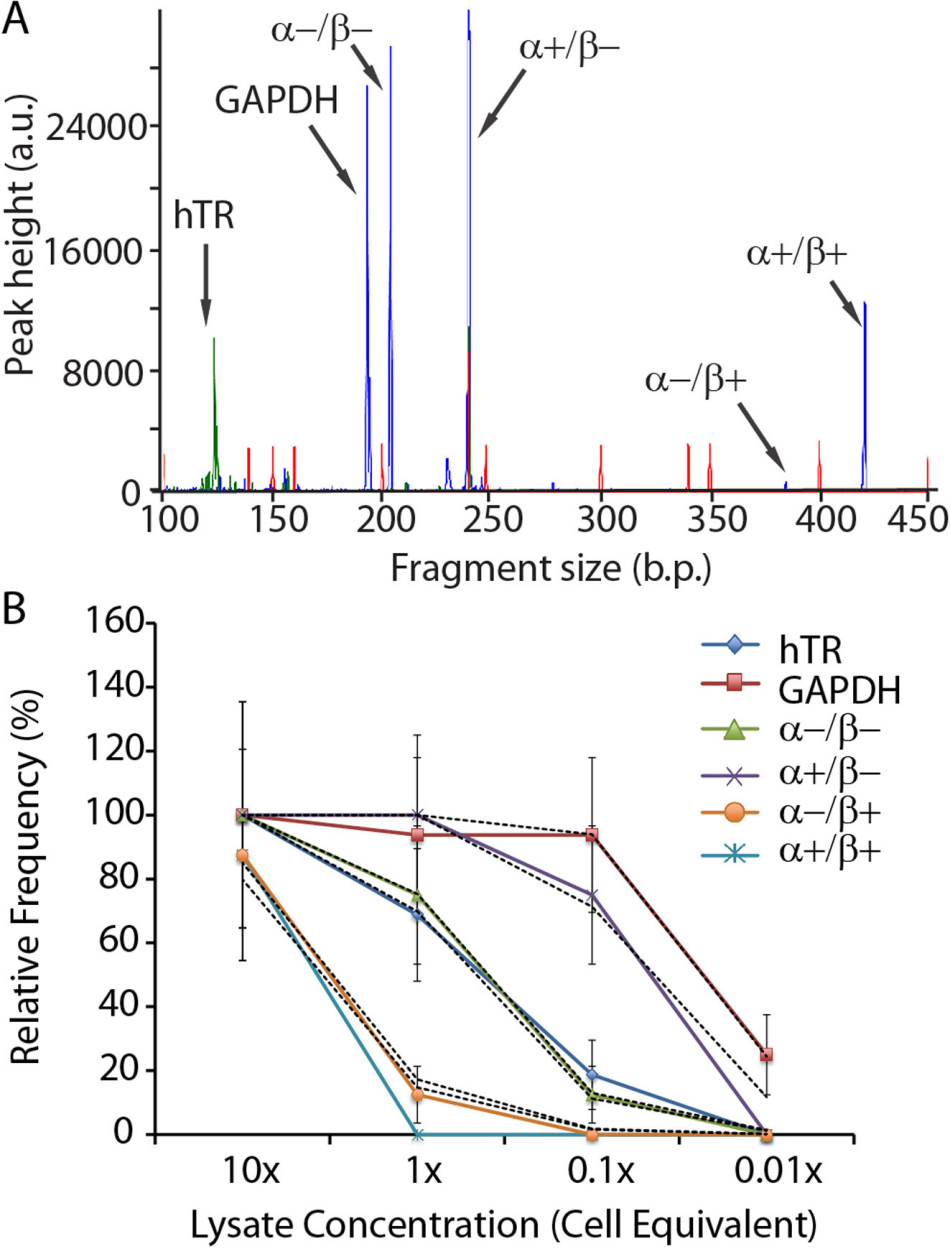
(**A**) Example of electrophoretic fragment analysis of multiplex RT-PCR from approximately 100 Jurkat cells used to simultaneously detect hTR, GAPDH and 4 potential hTERT splice variants at the α and β splice sites. Primers fluorescently labeled with FITC (blue peaks) and HEX (green peaks) enable accurate product determination and sensitive quantitation. The red peaks are an ROX-labeled size standard. (**B**) Plot of frequency of positive output reactions versus serial dilutions of Jurkat cell lysate (colored lines) and the least squares Poisson distribution fit (dashed black lines) for each target; close adherence to the Poisson distribution indicates single molecule detection sensitivity and allows estimation of copy number in each sample. The lysate amount equivalent to a single cell contained on average 1.4 α−/β−, 12.5 α+/β−, 0.16 α−/β+ and 0.19 α+/β+ hTERT copies per cell. Average GAPDH and hTR copy numbers could not be determined from this assay since the respective primer pairs were used at 10-fold lower concentrations compared to the hTERT primer pair in order to avoid amplification bias due to their very high native expression levels. *N* = 18 for each data point. Error bars are Poisson error estimates calculated from the number of positive reactions.

To simultaneously measure targets with a wide range of abundance levels in single cells, we developed and optimized a multiplex RT-PCR assay followed by hemi-nested PCR reamplification. A critical requirement was the ability to detect single molecules of the four hTERT α and β mRNA splice variants while simultaneously detecting hTR RNA despite its strong secondary structure and relatively high transcription level in cancer cells (6). The RT-PCR reagents chosen enable direct addition of single cells or colonies to the reactions in order to avoid lengthy RNA extraction steps, template degradation, and an associated decrease in sensitivity. Triton X-100 detergent lyses the cell membranes, while dithiothreitol (DTT) and an RNAse inhibitor stabilize the RNA molecules. GAPDH and hTR primer concentrations were decreased 10-fold versus hTERT primers to avoid overwhelming the reaction with these highly transcribed targets. Finally, the inclusion of 1M betaine as an isostabilizing agent and the implementation of a rapid cooling step during reverse transcription, allowed us to overcome the inhibitory effect of hTR secondary structure formation without affecting other targets. A second, hemi-nested PCR step following the initial reverse transcription and 15 cycles of highly specific PCR allowed for strong amplification of targets with additional specificity and low bias. In Figure 3A, the result of a bulk cell experiment with 100 Jurkat cells shows the fluorescently labeled amplicon electropherogram of hTR, GAPDH and the four telomerase splice variants.

We confirmed assay sensitivity and lack of target interaction by performing RT-PCR and reamplification on serial dilutions of a 10 Jurkat cells/μl lysate followed by readout using CE separation and automated analysis as described in the methods (*N* = 18 for each sample). This digital RT-PCR approach permitted a validation of our assay on actual cell samples while demonstrating the ability to detect single copies of RNA targets without interference from other targets in the multiplex reaction or off-target interference from other RNA. For digital PCR in general, demonstrating that assay results of serially diluted samples follow the Poisson distribution is a widely accepted means of demonstrating the ability to detect single molecule targets in a novel assay (18,19). Alternative approaches for demonstrating single molecule detection, including *in vitro* transcribed RNA spiking of reactions, would not provide information on interference from complex samples like cell lysate, which contains inhibitory factors as well as off-target RNA at large concentrations. It is important to note that the digital detection approach was only used for assay validation, while all subsequent data were obtained from an analog quantitation of capillary electrophoresis peaks.

For each target at each input concentration, the frequency of positive reactions is plotted in Figure 3B. For the various splice variants the frequency of positive reactions follows the Poisson distribution, indicating the ability to detect single molecules. Least squares fitting of the frequencies to the λ variable of the Poisson distribution, shown in dashed lines for each target in Figure 3B, permitted an estimate of the average number of hTERT splice variant molecules per cell: 1.4 α−/β−, 12.5 α+/β−, 0.16 α−/β+ and 0.19 α+/β+. This result agrees with a prior qPCR-derived estimate of 0.44 α+/β+ and 15 total hTERT mRNA copies per cell on average in HL60 promyelocytic leukemia cells, the most similar cell line tested by Yi *et al*. (7) Absolute GAPDH and hTR copy numbers could not be determined in this assay due to the significantly lower primer concentrations intentionally used to decrease the reverse transcription efficiency of those high-copy targets. Nevertheless, even those targets are detected with single molecule sensitivity with PCR once the RNA is reverse transcribed. Lastly, we confirmed the lack of significant competition between the assay targets by observing the correlation between expression of hTERT and hTR. The plot of hTR versus hTERT levels in Supplementary Figure S2 shows minimal correlation (*R*^2^ = 0.0046), indicating low amplification competition as expected from independent primer sets used with homogeneous templates. Furthermore, no bias was observed between the 4 possible hTERT splice variants, presumably due to using a single primer pair to amplify them.

### Single colony transcript abundance and splicing co-occurrence analysis

**Figure 4.**
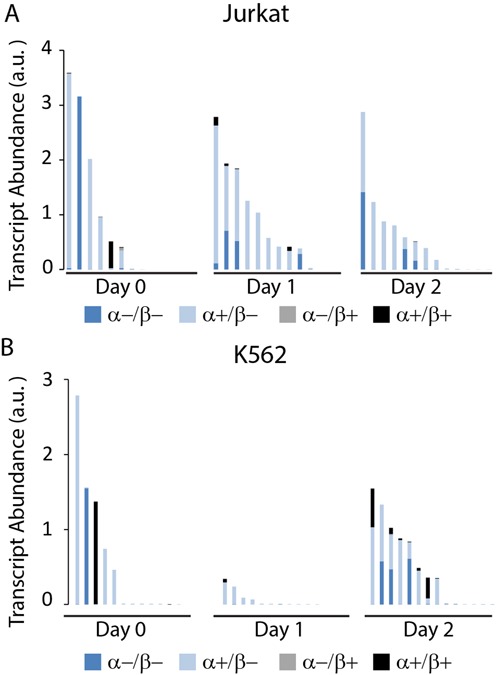
Single colony hTERT splice variant co-expression plots for (**A**) Jurkat and (**B**) K562 cells for days 0, 1 and 2. Each column represents results from one cell or colony. For each day, data are ordered by decreasing total hTERT transcript abundance levels. On day 0, when nearly all samples consist of single cells, hTERT consists of predominantly a single splice variant. Splice variants co-occur on other days presumably due to multiple cells present per sample, suggesting that a single cell expresses a single hTERT splice variant at any given time.

Jurkat and K562 lymphocyte cells were analyzed at one day intervals using the multiplex RT-PCR assay to investigate the co-occurrence of alternatively spliced hTERT mRNA variants and to examine changes in these patterns during colony growth. Figure 4 shows transcript abundance of single cells and colonies normalized per cell for Jurkat (A) and K562 cells (B) on each of the three days of culture ordered by total hTERT expression. On day 0, soon after encapsulation, colonies consisted almost exclusively of single cells, each of which exhibits a different hTERT transcript abundance but also each cell expresses primarily a single hTERT splice variant. On subsequent days, this trend is overtaken by increased co-occurrence of multiple splice variants, presumably due to the presence of multiple cells in each colony. Although detection bias cannot be completely excluded, this finding suggests that single cells are largely expressing one splice variant at a time. Absolute hTR levels per cell remained relatively stable during colony growth and were excluded for clarity. Further investigation is needed to illuminate the mechanisms involved in hTERT splicing regulation at the single cell level, whether due to epigenetic, paracrine signaling, or other means.

### Bimodality of hTERT abundance

**Figure 5.**
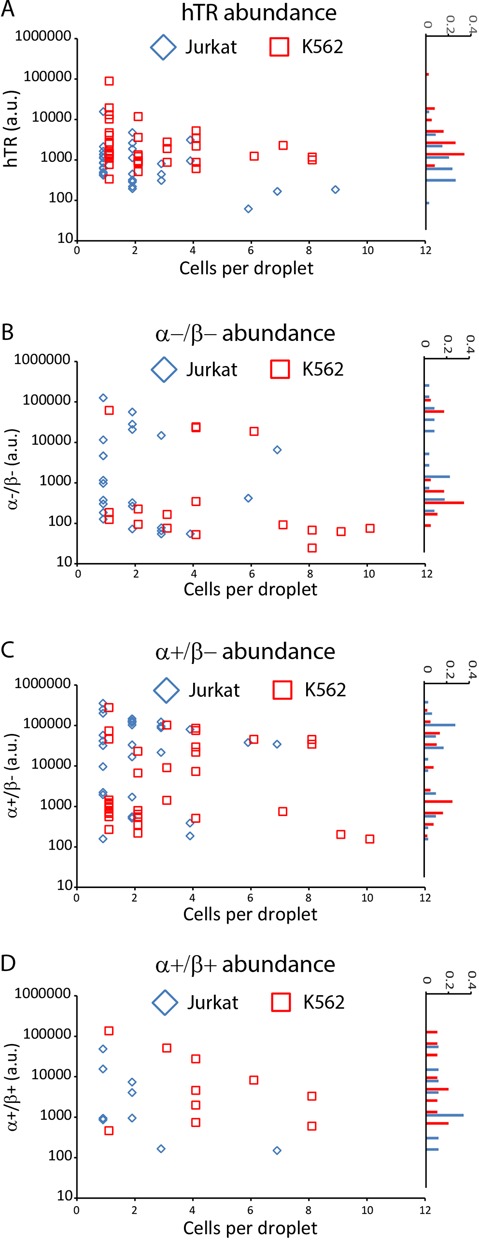
(**A**–**D**) Scatter plot of cell number per droplet versus transcript abundance for hTR and 3 hTERT variants (left graph in each panel) with corresponding histograms of transcript abundance frequencies (right graph in each panel) for Jurkat (blue diamonds) and K562 (red squares) cell lines. The α−/β+ variant was not expressed in a significant number of colonies and was omitted. The α−/β− and α+/β− splice variant abundances approach bimodal patterns in both cell types, though only the α+/β− splice variant in K562 cells is statistically significant (dip = 0.0823, *P* = 0.0424). The transcript abundances of hTR and the α+/β+ variant are not bimodal.

hTERT expression is overall more heterogeneous than hTR at the single cell level. This variability persists throughout colony expansion and, unlike hTR, does not trend toward the population average. Figure 5A shows that while there is significant heterogeneity in hTR transcript abundance in single cells, colonies of both Jurkat and K562 cells converge to a less variable population average with increasing colony size. Furthermore, single cell transcript abundance frequency follows an approximately normal distribution that is statistically not bimodal. In contrast, the α+/β− variant exhibits significant bimodal expression in the K562 cell line (dip = 0.0823, *P* = 0.0424), best observed in the histogram of Figure 5C, which translates into bimodal total hTERT abundance (dip = 0.0888, *P* = 0.0258). The α−/β− splice variant in K562 cells approaches bimodality, though the bimodality is not statistically significant (dip = 0.0906, *P* = 0.26). In Jurkat cells, both the α−/β− and α+/β− splice variants approach bimodality but do not attain statistical significance between day 1 and day 2 (dip = 0.0712, *P* = 0.472 and dip = 0.0563, *P* = 0.683, respectively). The α+/β+ full-length variant exhibits a negative correlation with colony size for both cell types, with a markedly more rapid decline for Jurkat cells compared to K562 cells.

### Curcumin modulates hTERT abundance and splicing

We sought to alter hTERT expression by exposing cells to sub-lethal curcumin concentrations in culture with the goal of observing mRNA expression and splicing modulation without a significant confounding effect caused by high apoptosis rates and other global changes. Curcumin has been shown to impact telomerase enzymatic activity through altered chaperone protein binding as well as expression levels (28–30). We cultured K562 cells in several curcumin concentrations to determine a sub-lethal dose that would not have a significant growth impact on cells. Supplementary Figure S3 summarizes the growth of K562 cells in bulk culture (A) and droplet cultures (B). In both droplet and bulk cultures, 10 μM curcumin significantly decreased growth rate, although growth remained positive. Growth rate differences between 1 μM curcumin and controls were not statistically significant on both days of culture (day 1: *P* = 0.218; day 2: *P* = 0.307). These results agree with prior assessments of curcumin effects on K562 cells, which found IC_50_ concentrations to be approximately 42 and 54 μM after 48 h exposure (31,32).

**Figure 6.**
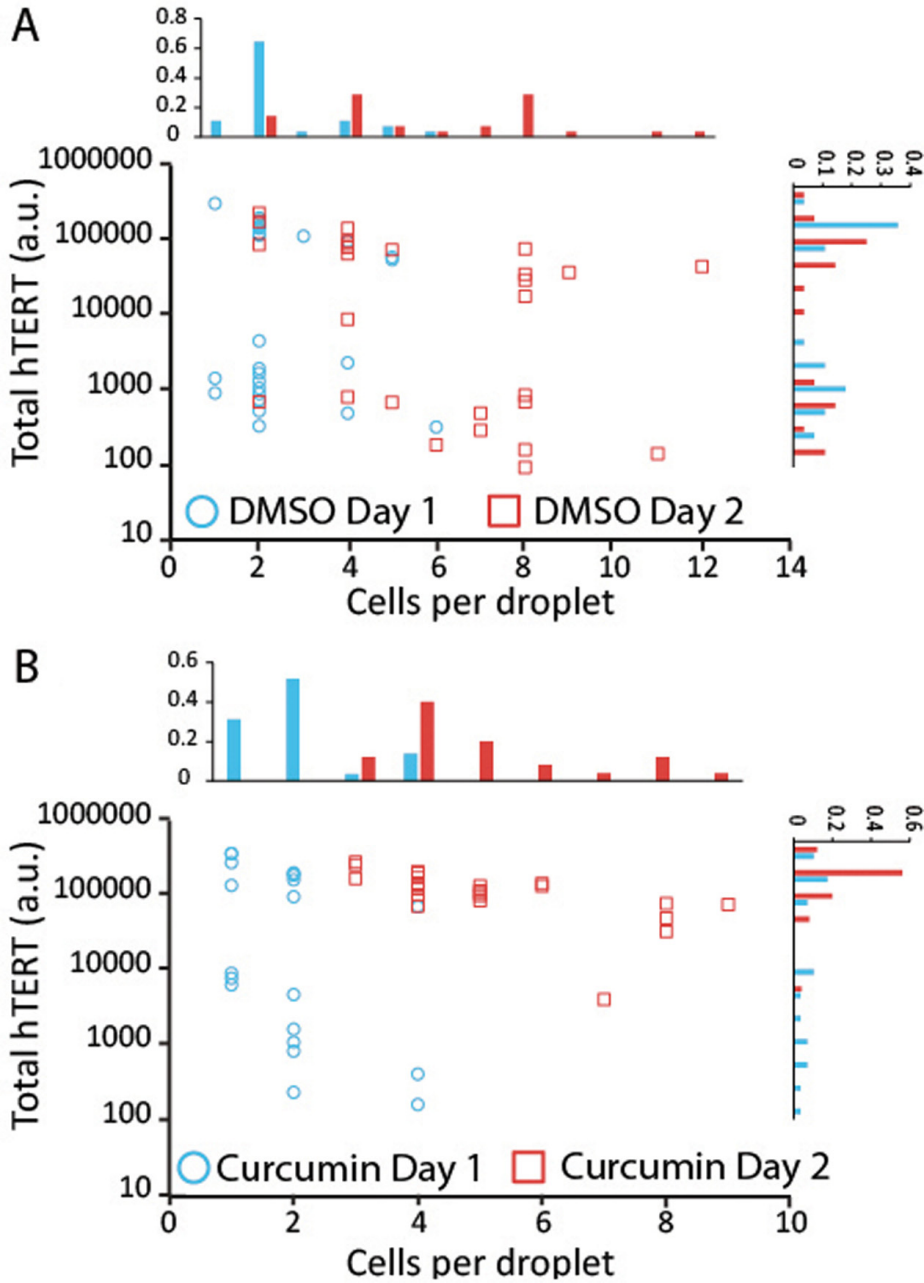
(**A**) Scatter plot of total hTERT transcript abundance versus cells per droplet for DMSO-treated control K562 cell cultures and associated normalized histograms of cells per droplets (top plot) and transcript abundance (right plot). Significant bimodal total hTERT transcript abundance can be seen for both day 1 and 2 (Day 1: dip = 0.136, *P* < 0.0001; Day 2: dip = 0.111, *P* = 0.0054). (**B**) Scatter plot of total hTERT transcript abundance versus cells per droplet for 1 μM curcumin-treated K562 cell cultures and associated normalized histograms of cells per droplets (top plot) and expression levels (right plot). A decrease in hTERT transcript abundance bimodality occurs on day 2 (Day 1: dip = 0.0995, *P* = 0.083; Day 2: dip = 0.0419, *P* = 0.993).

Figure 6 presents total hTERT abundance for control (A) and 1 μM curcumin-treated K562 cultures (B). On day 1, treated cells show nearly-bimodal total hTERT expression patterns similar to the strongly-bimodal controls, though the bimodality is statistically significant only for controls (Control: dip = 0.136, *P* < 0.0001; Curcumin: dip = 0.0995, *P* = 0.083). By the second day, however, curcumin treatment results in the disappearance of the low-hTERT population, while the control cells maintain bimodality (Control: dip = 0.111, *P* = 0.0054; Curcumin: dip = 0.0419, *P* = 0.993). The decreased bimodality, measured by the decrease in dip value, can be seen in the hTERT histograms. This is paralleled by decreased synchronization in cell growth.

**Figure 7.**
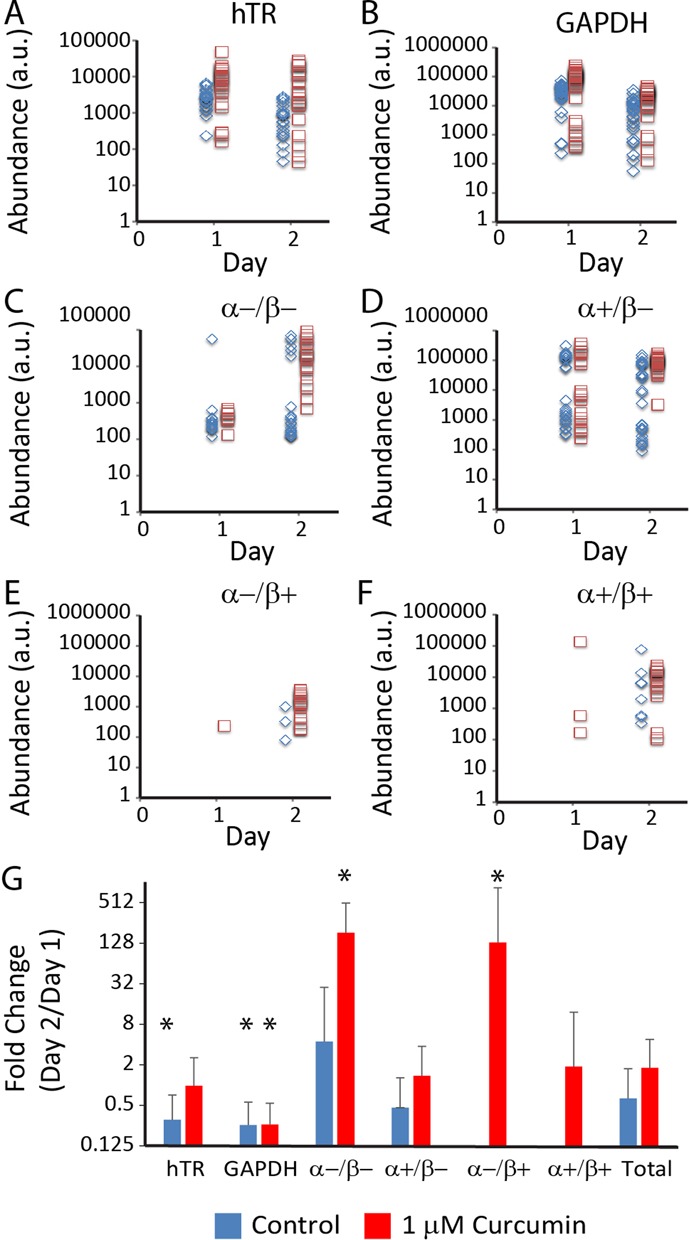
(**A**–**F**) Scatter plots of transcript abundance for all gene targets on days 1 and 2 for K562 cell cultures treated with DMSO (blue diamonds) and 1 μM curcumin (red squares). Curcumin treatment results in overall higher transcript abundance for all targets, but primarily increases abundance of both enzymatically-inhibitory α− splice variants, thereby reducing bimodality of total hTERT transcript abundance. (**G**) Graph showing change in mean transcript abundance from day 1 to day 2 for each target. Stars indicate statistically significant (*P* < 0.01) and biologically relevant (fold change > 2 or < 0.5) modulation of RNA levels between day 1 and day 2. Fold change values could not be calculated for α−/β+ and α+/β+ splice variants for control colonies and were omitted from the plot. The >100 fold increase in abundance of α− variants on day 2 suggests a strong impact of curcumin on α− splicing at sub-lethal concentrations.

Non-lethal curcumin exposure also alters hTERT splicing patterns. Figure 7 presents the transcript abundance of hTR, GAPDH, and the four hTERT splice variants for control and curcumin-treated colonies. While hTR and GAPDH transcript abundance changes only subtly in magnitude between day 1 and 2, curcumin has a significant impact on hTERT splicing and transcript abundance. The two α− splice variants (C and E) are significantly upregulated on day 2 compared to curcumin-treated cells on day 1 and compared to controls on both days. The α+/β− variant (D) is highly abundant for most treated colonies on day 2, thereby decreasing the degree of bimodality compared to treated colonies on day 1 (Day 1: dip = 0.0995, *P* = 0.0888; Day 2: dip = 0.0463, *P* = 0.962) and on both days for control cells (Day 1: dip = 0.136 *P* = 0.0002; Day 2: dip = 0.112, *P* = 0.0044). Unexpectedly, the α+/β+ full-length hTERT mRNA abundance is also upregulated following curcumin exposure, with treated cultures exhibiting a greater number of colonies containing the full length product compared to controls.

The strong upregulation of α− splicing is summarized in Figure 7G, which shows the fold change of mean expression levels of colonies from day 1 to day 2. Curcumin treatment results in a >100-fold statistically-significant increase in transcript abundance signal for the two α− splice variants, whereas β− splicing is not significantly affected. Control cells show only a subtle change in the hTERT variant levels between the two days. The α−/β+ and α+/β+ variants were not detected on day 1 in control cultures, and the fold change could not therefore be analyzed. However, the α−/β+ and α+/β+ transcript abundance differences between control and curcumin-treated colonies were not statistically significant (*P* = 0.161 and *P* = 0.119, respectively) on day 2 alone, suggesting that the control colonies behave similarly for these splice variants, albeit at lower expression magnitudes. GAPDH is downregulated approximately 3-fold for both controls and treated cells, while hTR decreases with a similar magnitude change only in control cells.

### Colony subculture

To investigate the persistence of splice variant modulation following curcumin treatment, we subcultured single colonies of 2–9 cells in 96 well culture plates with 50 μl of culture medium lacking curcumin. Ten colonies each were pipetted from curcumin-treated droplet cultures and controls on day 2, and wells were examined using microscopy to confirm the successful transfer of the entire colony and droplet lysis. Supplementary Figure S4A shows the success or failure of colonies for a given colony size. Both curcumin-treated and control colonies were well-represented and larger initial colony size resulted in more successful subcultures, but curcumin-treated colonies with fewer than 8 cells were much more likely to fail to grow. Twelve days following the initiation of subculture, 5-cell samples were analyzed using the multiplex RT-PCR assay. The results shown in Supplementary Figure S4B indicate the recovery of hTERT bimodality and splicing from modulation due to the initial curcumin treatment, suggesting that hTERT splicing and expression changes are reversible.

## DISCUSSION

Cellular environmental changes and stimuli, including sub-therapeutic drug dosing, can have a pronounced effect when measured at the single cell level that may not be possible to detect accurately in the bulk cell population. To enable analysis of subpopulations of “early responders,” we have developed a versatile droplet-based single cell culture method that enables unprecedented parallel monitoring of cell growth, relative transcript abundance analysis, response to drug treatment and subculturing of single-cell-derived colonies. By encapsulating single cells in nanoliter-scale droplets, individual cells are cultured at concentrations comparable to those of bulk cultures avoiding confounding effects of slow growth rates at non-native densities. The effective cell concentration experienced by cells is determined by the droplet volume, enabling highly accurate cell densities independent of the number of encapsulated cells. By segregating single cells into independent compartments, complex cell-cell signaling can be eliminated and selectively reintroduced using soluble factors or co-culture of defined cell numbers and types. Furthermore, the rapid droplet generation process uses simple hardware and avoids extensive pipetting and cell sorting, making the technique very accessible. Thus, culture droplets as described here provide a convenient new tool for generating, culturing and analyzing single cells for a wide range of applications, including multiparameter single cell analysis.

The practical utility of this single cell technique has been demonstrated by culturing single Jurkat and K562 cells in droplets to correlate cell growth with telomerase gene expression and hTERT splicing. When placed in 6 nl droplets, these cells undergo 2–4 divisions at a rate comparable to standard cell culture conditions. The resulting single cells and colonies are assayed for hTERT, hTR and GAPDH RNA using multiplexed RT-PCR with fluorescent primers and subsequent capillary electrophoresis detection to quantify transcript abundance and splice variant ratios. Cell lysate dilution experiments reveal that there are approximately 10–20 hTERT molecules per cell in the Jurkat cell line. Furthermore we find that the α+/β− splice variant in particular exhibits a high degree of transcript abundance bimodality independent of colony size, while the α+/β+ variant abundance slightly decreases with colony size. These results suggest that the full-length hTERT mRNA becomes downregulated as cells approach the droplet culture carrying capacity. In addition, the persistence of the α+/β− splice variant in larger colonies fits with previous studies that have correlated this splice variant with decreased telomerase activity (9,11,33). This result suggests that the ratio of full-length hTERT to other splice variants may be more biologically relevant than the abundance of a single variant alone.

The persistence of bimodality in the α+/β− splice variant despite colony growth supports the presence of bursting dynamics of hTERT gene expression, since the occasional expression of a particular hTERT splice variant by relatively few cells would give rise to persistent bimodality among small colonies. As the gene is transcribed on the order of tens of copies per cell, the gene bursting behavior seen in other studies could apply here as well (34,35). Depending on the frequency of transcription, relatively few cells should contain high levels of an expressed gene. Indeed, we observed single cells with predominantly single splice variants, with typically fewer than 25% of single cells containing relatively high levels of a particular splice variant. This low frequency and evidence of predominantly single splice variants per cell suggest that bursting behavior is extended to splice variants, with cells expressing bursts of single splice variants at a given time, though changes in mRNA degradation rates cannot be discounted.

This behavior may originate from the physical state of the hTERT DNA in the nucleus. Recent work has shown that chromosome architecture plays a dominant role in hTERT splicing, demonstrating the effect of accessible repetitive DNA in an hTERT minigene on alpha and beta splicing (36). In addition, there is strong evidence that gene expression burst intervals, including transcriptional regulation of hTERT, are closely tied to chromosomal architecture and nucleosome assembly and modifications (37,38). Taken together, these findings point toward single hTERT splice variant bursts in individual cells.

Treating cells with sub-lethal curcumin concentrations enabled us to observe changes in hTERT levels and splicing without the confounding effect of decreased growth rates. Although the resulting 100–1000-fold increase in total hTERT transcript abundance and decreased expression bimodality was surprising, these findings can be explained by earlier work showing that the α−/β+ and α+/β− variants are inhibitors of telomerase activity (11,12), and their upregulation may be counteracting the increase in full-length hTERT mRNA during translation. Our data suggest that the α−/β− variant also plays an inhibitory role. Increased active hTERT expression may be counteracted by a similar or greater upregulation of enzymatically-inhibitory alternative splice variants. In addition, studies showing a decrease in both hTERT abundance and telomerase activity following curcumin exposure used significantly higher curcumin concentrations that resulted in apoptotic pathway upregulation and decreased cell viability (29,30). Cui *et al*. tested 1 μM curcumin, among other concentrations, and found telomerase activity to decrease after 120 h but did not examine hTERT gene expression or transcript abundance (39).

Curcumin exposure also seems to slightly affect colony size by decreasing the number of very large colonies (>8 cells). This effect could be due to modulation of cell cycle; relatively synchronized daughter cell division, with most colonies having 1, 2, 4 or 8 cells, is evident in control cultures, while curcumin cell number frequencies have more intermediate colony sizes suggestive of unsychronized cell division. Indeed, curcumin has been shown to impact cell cycle through G2/M growth arrest in other cell lines (30).

Upregulation of hTERT may also be a telomerase activity-independent response to stress caused by curcumin exposure. Akiyama *et al*. demonstrated that K562 cells overexpressing full-length hTERT were less sensitive to apoptosis induced by serum deprivation and double strand DNA breaks (40). Listerman *et al*. found that overexpressing the α+/β− variant protected breast cancer cells from cisplatin-induced apoptosis *in vitro* (11). Our results could indicate a similar protective role for the alpha splice variant as well, though further validation, including an investigation of the effects of curcumin on cell cycle, is needed.

In future applications, dosing cells at sub-lethal concentrations and performing single cell analysis of gene expression could enhance our understanding of therapeutic effects of compounds during drug screens. Drug testing has progressed toward screening extremely low concentrations of candidate drugs in Phase 0 trials using microdosing, where pharmaceutical dynamics and kinetics can be estimated from subtherapeutic doses and drug-drug interactions can be predicted safely (41–43). However, microdosing requires highly sensitive mass spectroscopic approaches that may be difficult to scale to large sample sizes and reach broad adoption. Single cell droplet-based culture offers a new platform for drug screening that takes advantage of as opposed to masking cellular heterogeneity. Rather than waiting for whole-tissue effects to set in, low drug concentrations could be used to analyze effects on a subpopulation of cells and extrapolated to the bulk for higher concentrations. Furthermore, the effects on a subset of cells can be quite large if assayed at the single cell level, thereby providing a robust way to measure effects masked by the ensemble average. This method would have the advantage of faster turnaround times with the possibility of integrating RNA-seq for large scale, pathway-level analyses of a compound's biological effects. In addition, applying this approach to tumor therapy as part of a personalized medicine approach could help identify tumor cells that have the ability to escape chemotherapy for further subculture and analysis.

Although our current method's bottleneck consists of manually selecting colonies for analysis, in future work this step can be accelerated dramatically using microfluidic or conventional droplet sorting as demonstrated by several groups (44,45). Sorting can be simplified and the method extended to adherent cells by adding solid substrates, such as a bead or hydrogel, for attachment and colony sorting (17). Automated sorting would allow screening of thousands of colonies per experiment, limited only by the throughput of genetic analysis. Downstream integration of RNA-seq with single cell culture could dramatically increase the amount of information per cell, which could then be correlated with cell growth and other parameters. A similar single cell genomics approach has been used to understand pathways underlying the heterogeneous response of dendritic cells upon exposure to lipopolysaccharide (16). Alternatively, both cell culture and analysis could be performed entirely in droplets using splitting and merging of droplets with additional reagents (46). This approach benefits from high throughput and automation at the cost of greater device complexity. In addition, further validation of our method using synthetically transcribed RNA could be used to provide absolute transcript abundance quantitation for even greater depth of analysis of single cells.

In summary, the single cell culture method presented here offers a versatile platform for multiparameter analysis of various cell types, including the measurement of low-expression level genes. We show bimodal transcript abundance of hTERT splicing in single cells and demonstrate effects of sub-lethal curcumin doses on telomerase transcript abundance and splicing. Our findings suggest a significant role for hTERT alpha splicing in response to curcumin treatment. The combination of single cell culture and multiparameter analysis combined with low concentration drug dosing is a promising approach for understanding effects of drug treatment as part of early stage drug candidate screens and personalized medicine therapies that could otherwise be missed in bulk cell population analyses.

## SUPPLEMENTARY DATA

Supplementary Data are available at NAR online.

SUPPLEMENTARY DATA
