# Supplementary material for: Single cell measurement of telomerase expression and splicing using microfluidic emulsion cultures
Source: Nucleic Acids Res. 2015 Jul 21;43(16):e104. doi: 10.1093/nar/gkv477 (PMC4652743; doi:10.1093/nar/gkv477)
Supplement: SUPPLEMENTARY DATA [file supp_43_16_e104__index.html]

Single cell measurement of telomerase expression and splicing using microfluidic emulsion cultures — Single cell measurement of telomerase expression and splicing using microfluidic emulsion cultures — SUPPLEMENTARY DATA 

# Single cell measurement of telomerase expression and splicing using microfluidic emulsion cultures

## SUPPLEMENTARY DATA

- SUPPLEMENTARY DATA
