# Supplementary material for: Single cell measurement of telomerase expression and splicing using microfluidic emulsion cultures
Source: Nucleic Acids Res. 2015 Jul 21;43(16):e104. doi: 10.1093/nar/gkv477 (PMC4652743; doi:10.1093/nar/gkv477)
Supplement: SUPPLEMENTARY DATA [file supp_gkv477_nar-00404-met-g-2015-File009.docx]

**Table S1.** Primers used for RT-PCR and hemi-nested PCR reamplification. Melt points are shown for both RNA and DNA targets for the relevant RT-PCR primers (RNA T_m_/DNA T_m_), and all temperatures were calculated for the experimental conditions using Oligo Analyzer 3.1 (Integrated DNA Technologies).

| RT Primers | T_m_ (°C) | Sequence (5’🡺3’) |
| --- | --- | --- |
| FAM Rev-hTERT RT-PCR* | 64.5/67 | /56-FAM/CGCAAACAGCTTGTTCTCCATGTC |
| For-hTERT* | 63.5 | GCCTGAGCTGTACTTTGTCAA |
| Rev-GAPDH | 50.8/56.7 | CAAAGTTGTCATGGATGACC |
| Cy5 For-GAPDH RT-PCR | 61.3 | /5Cy5/AGTCCACTGGCGTCTTCAC |
| HEX Rev-hTR | 48.6/59.6 | /5HEX/TAGAATGAACGGTGGAAGGC |
| For-hTR RT-PCR | 65.5 | TTGCGGAGGGTGGGCCT |

| Hemi Nested Primers | T_m_ (°C) | Sequence (5’🡺3’) |
| --- | --- | --- |
| FAM Rev-hTERT Nested* | 66.7 | /56-FAM/AGGCTGCAGAGCAGCGTGGAGAGG |
| For-hTERT* | 62.6 | GCCTGAGCTGTACTTTGTCAA |
| Rev-GAPDH | 58.0 | CAAAGTTGTCATGGATGACC |
| FAM For-GAPDH Nested | 62.7 | /56-FAM/CCATGGAGAAGGCTGGGG |
| HEX Rev-hTR | 61.9 | /5HEX/TAGAATGAACGGTGGAAGGC |
| HEX For hTR Nested | 60.5 | /5HEX/CATTTTTTGTCTAACCCTAACTGAG |

*Adapted from Yi et al., 2001.(7)


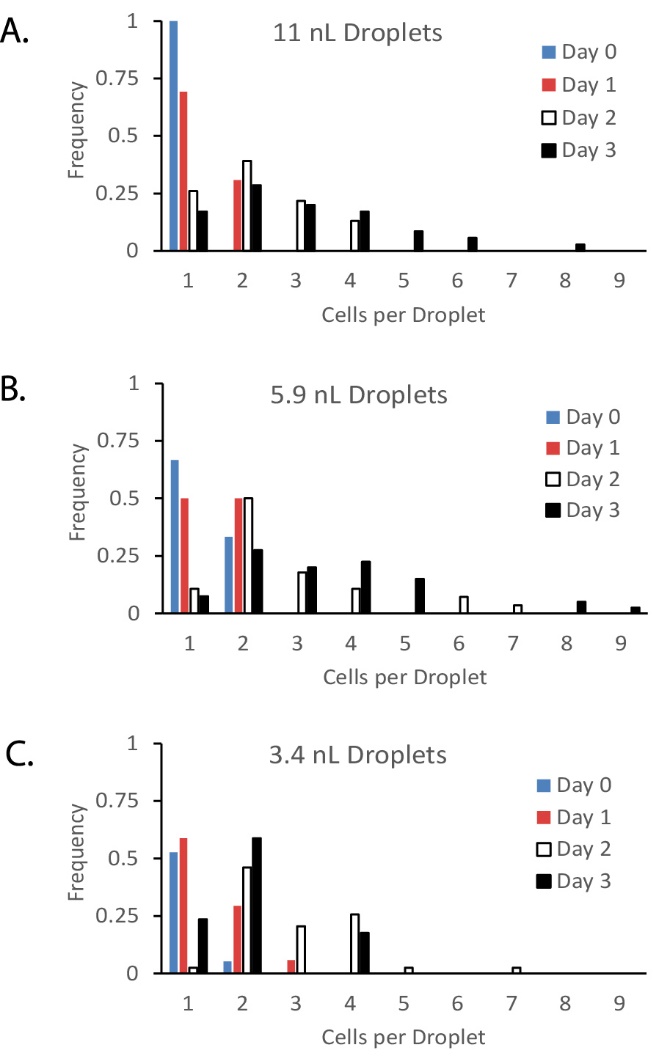


**Figure S1.** Histograms of colony size for days 0-3 in 11 nL, 5.9 nL, and 3.4 nL droplets. The smallest droplet volume results in rapid consumption of nutrients and cells show poor growth after day 2. The two larger droplet volumes provide sufficient nutrients to support cell growth.

**Figure S2.** Plot of log_10_-transformed hTR and hTERT transcript abundance obtained from serial dilutions of Jurkat cell lysate. To confirm that the RT-PCR assay would detect both hTR and hTERT RNA in an unbiased manner, Jurkat cell lysate was diluted over 4 orders of magnitude (10 to 0.01 cells/reaction) and assayed. With an estimated 10,000 hTR and 0-50 hTERT molecules per cell, each sample was still expected to contain at least 10 molecules of hTR on average, while hTERT molecules reached stochastic levels in nearly all samples. In an unbiased RT-PCR assay, the measured hTERT transcript abundance for single molecules should not vary with changes in hTR concentration. As expected from a reaction with minimal primer competition, no significant correlation between hTR and hTERT was observed across the wide range of target concentrations, indicating minimal assay bias over a relevant hTR and hTERT concentration range. Each point represents a separate RT-PCR reaction.

A.

B.

**Figure S3.** Comparison of growth rates among two curcumin concentrations and a DMSO control reveals a significant growth impact of 10 μM curcumin on cell growth and no significant effect between 0 μM and 1 μM curcumin. The results demonstrate that cellular drug response in droplets parallels that of traditional bulk cultures.


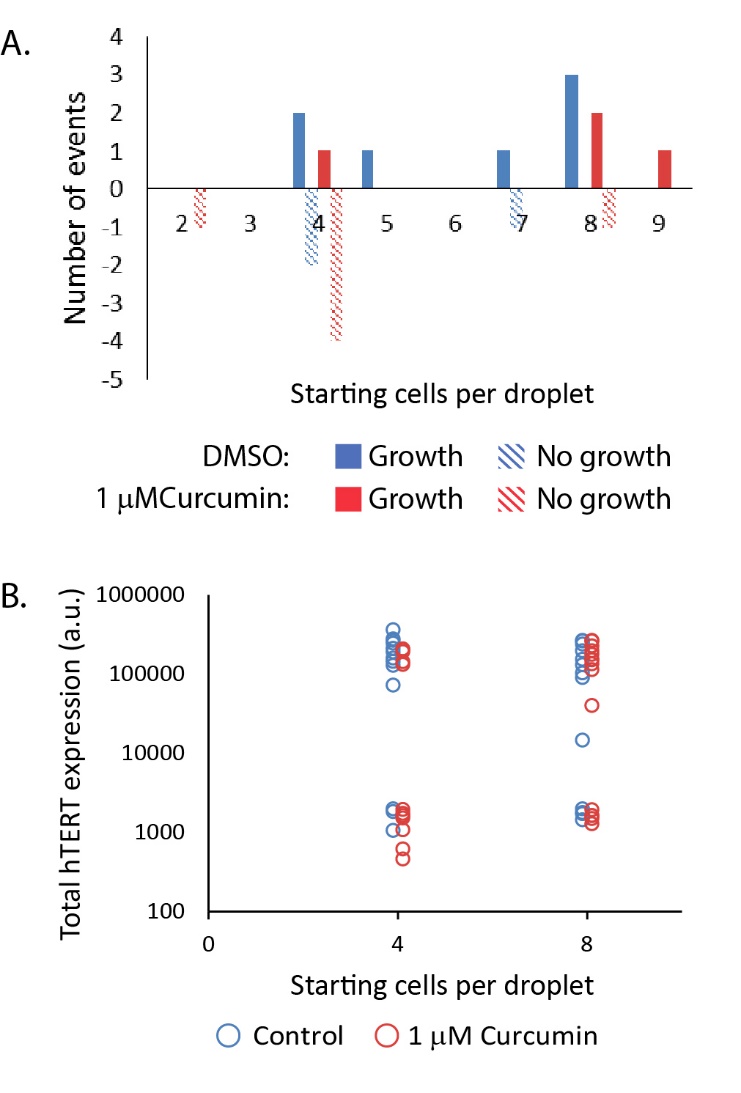


**Figure S4.** Subculture of droplet colonies for downstream analysis. (A) Frequency plot of survival of K562 colonies following subculture into wells containing 50 μL medium, with successful and failed subcultures represented by positive and negative values, respectively. Faster growing colonies (7-9 cells per droplet) have a greater probability of survival in subculture than slower growing colonies (2-5 cells per droplet). (B) Expression data with each point representing ~5-cell samples for 4-cell and 8-cell colonies each taken from control or curcumin-treated cells shows a reversion of total hTERT expression to the original bimodal pattern after two weeks of culture in curcumin-free medium.
